# Association of Khat and alcohol use with HIV infection and age at first sexual initiation among youths visiting HIV testing and counseling centers in Gamo-Gofa Zone, South West Ethiopia

**DOI:** 10.1186/1472-698X-13-10

**Published:** 2013-02-02

**Authors:** Marelign Tilahun Malaju, Gistane Ayele Asale

**Affiliations:** 1 Department of Public Health, College of Medicine and Health Sciences, Arba-Minch University, P.O. Box: 21, Arba-Minch, Ethiopia

## Abstract

**Background:**

HIV/AIDS is the major problem and an obstacle to both the health and development of people in Ethiopia today. It is also indicated that the use of substances have dramatically increased despite the serious concern about HIV infection.

**Methods:**

Unmatched case control study was conducted in South West Ethiopia using a sample of 105 cases and 305 controls. Multivariate logistic regression was used to assess the degree of association between dependent and independent variables.

**Result:**

HIV infection was positively associated with being in the age of 20 – 24 years [OR & (95% CI) = 2.892 (1.266, 6.607)], being female [OR & (95% CI) = 2.013 (1.061, 3.822)], alcohol use [OR & (95% CI) = 5.883 (3.034, 11.408)], having no education [OR & (95% CI) = 3.193 (1.523, 6.695)] and primary education level [OR & (95% CI) = 3.160 (1.351, 7.388)]. Early sexual initiation was also positively associated with being not employed Adj. HR & (95% CI) = 7.372 (1.455, 37.357)], not having comprehensive knowledge on HIV/AIDS [Adj. HR & (95% CI) = 8.247 (2.121, 32.067)], alcohol use [Adj. HR & (95% CI) = 3.815 (1.315, 11.070)] and khat use [Adj. HR & (95% CI) = 7.241 (1.871, 28.016)].

**Conclusion:**

Strategies should be designed to control the use of alcohol and khat which were found to be predictors of HIV infection and early sexual initiation in this study.

## Background

History of Substance drug abuse is as old as history of mankind. Human beings have been using the different parts of plants as medicine for relieving different health conditions and also as mediators in different religious and cultural ceremonies. Until 1950s, drug abuse was uncommon phenomenon. Today, there is an estimated 190 million drug abuser around the globe, which accounts for 3.1% of the world population or 4.3% of the population aged 15 years and above. Recent trends indicate that the use of substances have dramatically increased particularly in developing countries [1,2].

The use of khat leaves or alcohol are believed to alter one’s moods or emotional state either through the sustained release or inhibition of neurotransmitters, thereby enhancing or dampening the response of the individual. Effects of khat on the chewer include increased levels of energy, increased self-esteem, euphoria, increased libido, excitement, and increased proclivity for social interaction [3-5]. The use of alcohol among adolescents can also be harmful, leading to decreased academic performance, increased risk of contracting HIV, and other sexually transmitted diseases, or other psychiatric disorders such as lethargy, hopelessness, and insomnia [6]. Most people whose thinking is warped by continued substance use may not be able to see the harm resulting from their actions. Thus, there has been a strong linkage between substance use and casual or unsafe sexual practice despite the serious concern about HIV infection [3].

Concurrent with the HIV/AIDS pandemic, many countries within sub-Saharan Africa exhibit very high levels of alcohol consumption, one of the most prevalent behavioral risk factors implicated in the transmission of HIV and other STDs [6]. Of the 20 countries in Africa identified by the World Health Organization (WHO) with very high levels of child and adult mortality, it was estimated that for 2000 the total alcohol consumption per adult was 7.1 liters of absolute alcohol [7].

Khat is widely consumed among the youth of Ethiopia as shown by several prevalence studies. Insomnia is a common problem associated with the use of khat which prompts the chewer to use/abuse sedatives and to indulge in alcohol as a means of overcoming the side effect and hence the risk of exposure to HIV would be increased under such heavy influence of a combination of drugs [8,9]. However, the association of khat and alcohol use with HIV infection and early sexual initiation is not well studied in Ethiopia and particularly among youths in the present study area. Therefore, the main aim of this study was to determine the association of khat and alcohol use with HIV infection and early sexual initiation among youths in south west Ethiopia.

## Methods

A case–control study to determine the association of substance abuse with HIV infection and early sexual initiation among youths visiting HIV counseling and testing centers was conducted from January 23 -April 25, 2012 in Gamo-Gofa, South West Ethiopia. Gamo-Gofa is located about 505 km south west from Addis Ababa, the capital of Ethiopia. According to the 2007 census result it has a population of 1,595,570 and of this 794,485 were males and 801,085 were females. There are three hospitals and 68 health centers offering health care services for the total population. The study population was all youths attending HIV testing and counseling centers in public health facilities during the data collection period.

### Sampling procedure

Three hospitals and health centers offering HIV testing and counseling service in Gamo-Gofa zone was included in the study. Stratified sampling technique was used to select the study units in each health institution. Based on the number of customers who visited each health institution during the previous one year (annual report of each health institution), proportional allocation of the total sample size was carried out to attain the required sample size in each health institution.

The classification of cases and controls were based on sero status of those people tested for HIV. Those people who are found to be positive for HIV test were designated as cases and those who are found to be negative for HIV test were designated as controls. Youths who were 15–24 years and those who came for HIV counseling and testing purpose were included in the study and those who are unable to communicate were excluded from the study.

### Sample size determination

A sample size of 105 cases and 305 controls were determined using the methods of “difference between population proportions” with 80% power, 95% confidence level, a ratio of cases to controls being 1:3 and by assuming the proportion of khat use among controls is 41% which is taken from a previous study conducted in other parts of Ethiopia [3].Non-response rate in this study was estimated to be 10%, and hence an overall sample size of 410 youths were recruited in the study.

### Data collection and quality control

Data were collected during the pre test counseling and before the sero-status of the individuals was known. The collection of data continued until the required number of cases was identified. All HIV sero-negative individuals during the same period were included as controls. Data were collected using structured questionnaires. Data collectors were counselors from the respective health institutions who were trained on the procedures. The designed questionnaire was translated first into the local/national language (Amharic) and back translated to English to ensure its consistency. The questionnaires were pretested in similar settings two weeks before the actual data collection. The collected data were checked for completeness and consistency by a supervisor and the principal investigator on daily basis. Any error or ambiguity and incompleteness were corrected before the individual was told the test result in his/her visit for the post test counseling. HIV sero-status (HIV sero-positivity or sero-negativity) and age at first sexual initiation were considered as the outcome or dependent variable, while socio demographic variables (age, sex, residence, religion, ethnicity, income, occupation, education level), comprehensive knowledge on HIV/AIDS and risky behaviors (number of sexual partners, use of condom, peer pressure, viewing pornographic materials, khat use and alcohol use) were considered as independent variables.

### Operational definition

Comprehensive knowledge on HIV/AIDS was measured by ability to identify the two important prevention ways (being faithful and condom use), being aware that a healthy-looking person can have HIV and reject the two locally common misconceptions about HIV transmission (mosquito bite and sharing food). A study participant was considered as ever drinker if he/she responds yes to the question ‘Have you ever drunk alcohol in your life?’ Then follow up questions were employed to collect information such as drinking in the past one year and frequency of drinking. Current alcohol use is defined as use of alcohol at least once during the past 30 days before the survey. Participants were also considered as ever chewer if he/she responds yes to the question ‘Have you ever chewed khat in your life?’ Then follow up questions were employed to collect information such as khat chewing in the past one year and frequency of chewing. Current khat use is defined as use of khat at least once during the past 30 days before the survey and these operational definitions were adopted from the Ethiopian demographic and health survey 2011.

### D**ata processing and analysis**

Data were entered and analyzed using SPSS software version 16. Descriptive statistics such as frequencies and proportion was used to describe the study population in relation to relevant variables. Multivariate logistic regression and Cox-regression were utilized to assess the presence and degree of association between dependent and independent variables.

### Ethical consideration

Ethical clearance was obtained from ethical review board of Arba-Minch University and permission to conduct the study in each health facility was secured from the respective health institutions in Gamo-Gofa. Verbal informed consent from each study participants was obtained after clear explanation about the purpose of the study and written consent from the parents or legal guardians of participants less than 18 years old was obtained to participate in the study. Data collectors were HIV counselors from the respective health institutions in order to insure the confidentiality of the study participants’ HIV sero-status.

## Results

Out of the total 410 youths recruited, 405 of them participated actively in this study making the response rate of 98.8%. About 301(74.3%) of the study population were from urban areas while the rest 104 (25.7%) were from rural part of the study area. Two hundred seventy six respondents (68.1%) were never married followed by those who were married 99(24.4%) and the majority 299 (73.8) of them had education level of secondary and above, followed by those who have no education 59(14.6%).

Most of the respondents 197 (48.6%) were student by occupation followed by government employed 73(18%). Majority 183(45.3%) of them were Gamo by ethnicity followed by Gofa 114 (28.2) and 221(54.6%) were followers of orthodox Christianity followed by protestant 158 (39.0). Majority of the study participants 294(72.6%) were in the age of 20–24 years with mean (±SD) age of 20.57(±2.59) and most 243 (60.0) of them have monthly income of <= 450 Ethiopian currency ($ 25) (Table 1).

**Table 1:** Socio-demographic characteristics of youths visiting HIV testing and counseling centers in Gamo-Gofa, South West Ethiopia, 2012

**Variables**	**Cases: n (%)**	**Controls: n (%)**	**Total: n (%)**
**Age** [Mean(±SD)=20.57(±2.59)]			
15–19	13(3.2)	98(24.2)	111(27.4)
20–24	91(22.5)	203(50.1)	294(72.6)
**Sex**			
Male	39(9.6)	147(36.3)	186(45.9)
Female	65(16.0)	154(38.0)	219(54.1)
**Residence**			
Urban	76(18.8)	225(55.6)	301(74.3)
Rural	28(6.9)	76(18.8)	104(25.7)
**Ethnic group**			
Gamo	52(12.9)	131(32.4)	183(45.3)
Gofa	28(6.9)	86(21.3)	114(28.2)
Amhara	10(2.5)	31(7.7)	41(10.1)
Others*	14(3.5)	52(12.9)	66(16.3)
**Religion**			
Orthodox Christian	57 (14.1)	164 (40.5)	221 (54.6)
Muslim	8 (2.0)	10 (2.5)	18 (4.4)
Protestant	36 (8.9)	122 (30.1)	158 (39.0)
Others**	3(0.7)	5(1.2)	8(2.0)
**Education**			
No education	30 (7.4)	29 (7.2)	59 (14.6)
Primary Education	19 (4.7)	28 (6.9)	47 (11.6)
Secondary & above	55 (13.6)	244 (60.2)	299 (73.8)
**Occupation**			
Government employed	13 (3.2)	60 (14.8)	73 (18.0)
Merchant	22 (5.4)	28 (6.9)	50 (12.3)
House wife	14 (3.5)	17 (4.2)	31 (7.7)
Daily Laborer	27 (6.7)	27 (6.7)	54 (13.3)
Student	28 (6.9)	169 (41.7)	197 (48.6)
**Marital Status**			
Never married	43 (10.6)	233 (57.5)	276 (68.1)
Married/living together	42 (10.4)	57 (14.1)	99 (24.4)
Divorced/separated/ widowed	19 (4.7)	11 (2.7)	30 (7.4)
**Monthly income**			
<= 450 Ethiopian currency ( <= $ 25 )	65 (16.0)	178 (44.0)	243 (60.0)
451 – 999 Ethiopian currency ( $ 25.1 - $ 55.5 )	24 (5.9)	48 (11.9)	72 (17.8)
>=1000 Ethiopian currency ( >= $ 55.6 )	15(3.7)	75(18.5)	90(22.2)

* Wolayta, Konso, Gurage, Oromo. ** Catholic, Adventist, Only Jesus.

### Distribution of the study participants with respect to sexual and non-sexual behaviors

Ninety four percent of cases and sixty four percent of controls have ever had sex and 61.6% of first sex was unplanned among cases. The median age of sexual initiation in this study was 17 years (IQR= 4.0). The proportion of alcohol use was higher 50(47.6) among cases than controls 78(26.0%) and the proportion of khat use is more than twice as high among the cases as in the controls, 35(33.3%) versus 47(15.7) Table 2).

**Table 2:** Sexual and non-sexual behaviors of youths visiting HIV testing and counseling centers in Gamo-Gofa, South West, Ethiopia, 2012

**Variables**	**Cases: n = 105**	**Controls: n = 300**
**Ever had sex**		
Yes	99(94.3)	193(64.0)
No	6(5.7)	108(36.0)
**Age at first sex **[Median(±IQR)=17.0(±4.0)]		
< 18 years	63(63.6)	103(53.4)
>= 18 years	36(36.4)	90(46.6)
**First sex was:**		
Planned	38(38.4)	100(51.8)
Unexpected	61(61.6)	93(48.2)
**Alcohol use**		
Yes	50(47.6)	78(26.0)
No	55(52.4)	221(74.0)
**Khat use**		
Yes	35(33.3)	47(15.7)
No	70(66.7)	253(84.3)

### Differences of the study participants with respect to age at first sexual initiation

**Figure 1:**
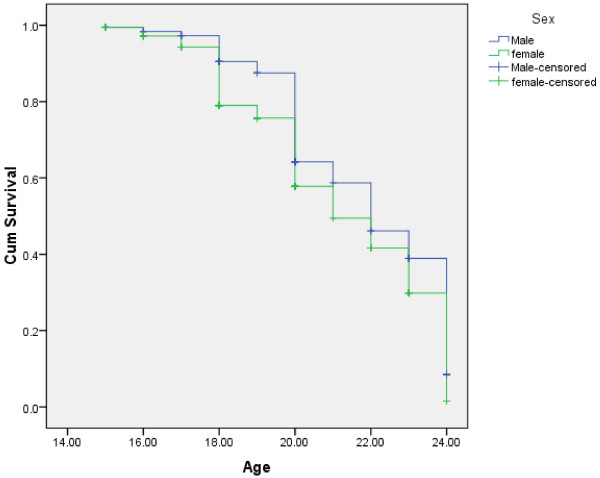
Kaplan-Meier indicating age at sexual initiation among male and female youths in Gamo-Gofa, South West Ethiopia (Log Rank chi-square = 6.85, p-value = 0.009).

**Figure 2:**
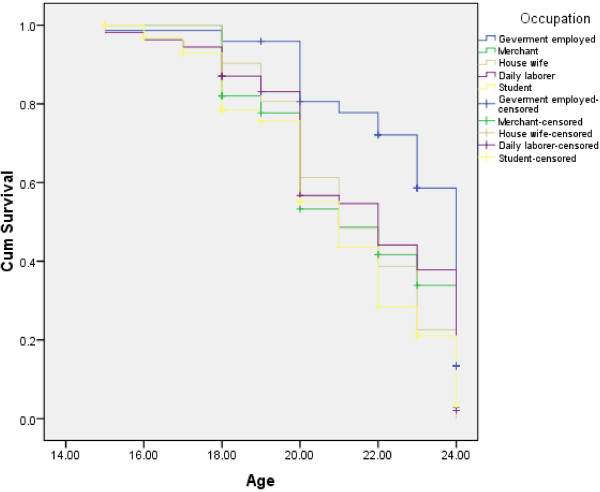
Kaplan-Meier indicating age at sexual initiation among youths with respect to occupation in Gamo-Gofa, South West Ethiopia (Log Rank chi-square = 33.12, p-value = 0.001).

**Figure 3:**
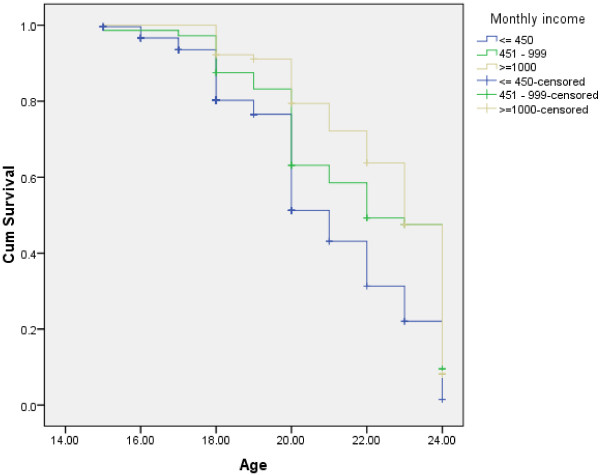
Kaplan-Meier indicating age at sexual initiation among youths with respect to monthly income in Gamo-Gofa, South West Ethiopia (Log Rank chi-square = 28.07, p-value = 0.001).

**Figure 4:**
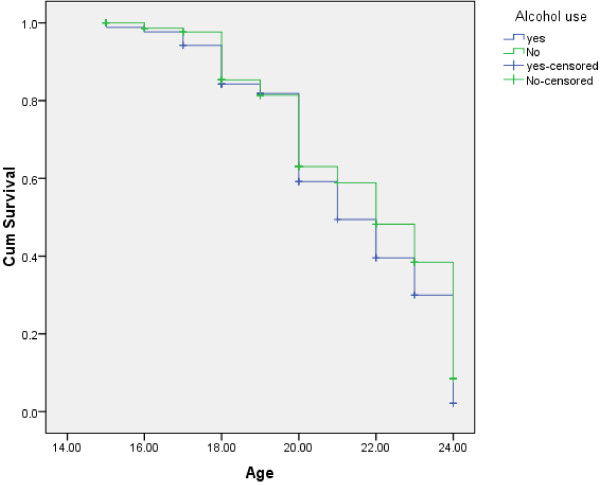
Kaplan-Meier indicating age at sexual initiation among youths with respect to alcohol use in Gamo-Gofa, South West Ethiopia (Log Rank chi-square = 3.75, p-value = 0.05).

As shown in Figure 1, females were found to initiate sexual intercourse at earlier stage than male respondents (log rank test having chi-square value = 6.85 and p-value = 0.009). With respect to occupation, students, daily laborers and merchants were found to initiate sexual intercourse at earlier stage than government employed (log rank test having chi-square value = 33.12 and p-value = 0.001) Figure 2. Compared to those with monthly income of >= 1000 Ethiopian currency or ( $ 55.6 ), those with monthly income of <= 450 Ethiopian currency or ( $ 25 ) were found to initiate sexual intercourse at earlier stage (Figure 3). Those respondents who ever had used alcohol were also found to initiate sexual intercourse at earlier stage than those who didn’t use alcohol (Figure 4).

### Predictors of early sexual initiation among youths in gamo-gofa, south west Ethiopia

In order to measure the association of age at first sexual initiation with a number of explanatory variables, crude and adjusted hazard ratio (HR) with 95% CI were employed and the result is given in Table 3. Compared to youths who are employed, those who are not employed were 7.372 times [Adj. HR & (95%CI) = 7.372 (1.455, 37.357)] as likely to initiate sexual intercourse before 18 years of age in Gamo-Gofa, South West Ethiopia. Youths who do not have comprehensive knowledge on HIV/AIDS were 8.247 times [Adj. HR & (95%CI) = 8.247 (2.121, 32.067)] more likely to initiate sexual intercourse before 18 years of age than those who do have. Compared to youths who do not use alcohol, those who are alcohol users were 3.815 times [Adj. HR & (95%CI) = 3.815 (1.315, 11.070)] as likely to initiate sexual intercourse before 18 years of age. In like manner, Khat users were 7.241 times [Adj. HR & (95%CI) = 7.241 (1.871, 28.016)] more likely to initiate sexual intercourse before 18 years of age than their counterparts. A statistically significant association was not found between age at sexual initiation and frequency of khat and alcohol use.

**Table 3:** Predictors of early sexual initiation among youths (Crude & adjusted HR) in Gamo-Gofa, South West Ethiopia, 2012

**Explanatory variables**	**Age at sexual initiation**		**Crude HR (95% CI)**	**Adjusted HR (95% CI)**	**P-value**
	**Early initiation < 18 years(1)**	**Late initiation >= 18 years(0)**			
**Employed**					0.016
Yes	72	66	1.00	1.00	
No	93	60	1.161 (0.853, 1.579)	7.372 (1.455, 37.357)	
**Have comprehensive knowledge on HIV**					0.002
Yes	80	73	1.00	1.00	
No	85	53	1.256	8.247(2.121, 32.067)	
**Alcohol use**					0.014
Yes	65	38	1.329(0.972, 1.817)	3.815(1.315, 11.070)	
No	100	88	1.00	1.00	
**Khat use**					0.004
Yes	42	29	1.082(0.762, 1.537)	7.241(1.871, 28.016)	
No	122	96	1.00	1.00	

### Factors associated with HIV infection among youths in gamo-gofa, south west Ethiopia

In order to measure the association of HIV infection with a number of explanatory variables, crude and adjusted odds ratio with 95% CI were applied and the result is given in Table 4. Compared to youths who are in the age range of 15–19 years, those who are in 20–24 years of age were 2.892 times [OR & (95% CI) = 2.892 (1.266, 6.607)] more likely to be infected with HIV. Female youths were about 2.013 times [OR & (95% CI) = 2.013 (1.061, 3.822)] more likely to be infected with HIV than males in Gamo-Gofa, South West Ethiopia. Compared to youths with education level of secondary and above, those with no education [OR & (95% CI) = 3.193 (1.523, 6.695)] and primary education level [OR & (95% CI) = 3.160 (1.351, 7.388)] were more likely to be infected with HIV. Alcohol users were 5.883 times [OR & (95% CI) = 5.883 (3.034, 11.408)] more likely to be infected with HIV than their counterparts. Likewise, compared to never married youths, those who are divorced/widowed/separated [OR & (95% CI) = 5.303 (1.922, 14.634)] and married/living together [OR & (95% CI) = 2.544 (1.278, 5.065)] were more likely to have HIV positive test result. But Frequency of khat and alcohol use was found to be non-significant.

**Table 4:** Factors associated with HIV infection among youths (Crude & adjusted OR) in Gamo-Gofa, South West Ethiopia, 2012

**Explanatory variable**	**HIV sero-status**		**Crude OR (95% CI)**	**Adjusted OR (95% CI)**	**P-value**
	**HIV positive**	**HIV negative**			
**Age**					0.012
15–19	13	98	1.00	1.00	
20–24	91	203	3.379(1.801, 6.340)	2.892(1.266, 6.607)	
**Sex**					0.032
Male	39	147	1.00	1.00	
Female	65	154	1.591(1.008, 2.512)	2.013(1.061, 3.822)	
**Education level**					0.002
No Education	30	29	4.589(2.548, 8.265)	3.193(1.523, 6.695)	
Primary education	19	28	3.010(1.569, 5.777)	3.160 (1.351, 7.388)	
Secondary & above	55	244	1.00	1.00	
**Marital status**					0.002
Never married	43	233	1.00	1.00	
Married/living together	42	57	3.993(2.387, 6.679)	2.544(1.278, 5.065)	
Divorced/separated/ widowed	19	11	9.359(4.161, 21.054)	5.303(1.922, 14.634)	
**Alcohol use**					<0.001
Yes	50	53	2.736(1.655, 4.523)	5.883(3.034, 11.408)	
No	50	145	1.00	1.00	

## Discussion

The median age of first sexual initiation (17 years) in this study is slightly higher than previous studies done in other parts of Ethiopia; in Dessie (16.8 years), in Kolladiba (15 years), in Gojam (13.5 years) and in Butajira (16 years) [10-13]. The difference may be explained by the decrease in early marriage which was the main reason for early sexual initiation in rural youths [12] due to the recently endorsed family law [14]. Another possible explanation could be that AIDS-related campaigns to delay first sexual intercourse may have had an inhibiting effect.

In this study alcohol users were almost four times more at risk to initiate sexual intercourse earlier than those who didn’t use alcohol. This is similar to earlier findings where alcohol users were two times and three times more likely to initiate sexual intercourse earlier that non-users in Dessie town and Butajira town respectively [10,11].

Youths who are khat users were also found to be seven times more likely to initiate sexual intercourse earlier than their counterparts. The association of khat use with sexual initiation in this study is stronger than the result obtained from other studies in Dessie town and Butajira where khat chewers were three times and two times at higher risk to initiate sexual intercourse earlier than non chewers in Dessie and Butajira respectively [10,11]. This difference might be due to the fact that our study is conducted in urban areas and the studies conducted in Dessie and Butajira are in rural areas indicating the difference of age at sexual initiation and khat utilization in rural and urban setting. The possible explanation for the association of khat use with sexual initiation in our study could be due to loss of track of mind induced by khat chewing. During the hypo manic phase, chewers may not be capable of rational judgment and they also may not be able to predict the serious consequences of their actions. Thus, the chewers could walk into the most dangerous situations feeling that there is no danger and being unaware of the possible dangers to their lives or well-being, they get motivated to have unplanned and early sexual initiation.

The present study also showed that unemployed youths were almost seven times more at risk to practice early sexual initiation than those who are employed. The higher risk of early sexual initiation among unemployed youths in our study raises our suspicion of the use of alcohol and khat by unemployed youths which predisposed them to have early sexual initiation. This possible explanation is supported by a study in Ethiopia in which the most frequent substance abusers were jobless youths and street children [15]. The 2001 Ministry of Health, Department of Pharmacy report also indicates that substance abuse is exacerbated by lack of employment opportunities and general feelings of hopelessness.

Viewing pornographic materials were not associated with early sexual initiation in this study which contrasts with previous study in Dessie town where youths viewing pornographic materials were 2.9 times more likely to initiate sexual intercourse earlier than their counterparts [10]. This might be due to few numbers of respondents who acknowledged viewing pornographic materials in our study populations or could be under-reporting of viewing pornographic materials because of social desirability bias or recall bias.

In this study the prevalence of Khat use 35 (33.3%) and alcohol use 50(47.6%) is found to be higher among HIV positive youths than their counterparts. This finding is similar with the result obtained from another study in Addis Ababa, where khat use (31.7%) and alcohol use (55.1%) were found to be higher among HIV positives [16].

Drug abuse has been incriminated as a potential exposure factor to HIV/AIDS by causing loss of inhibition and involvement in risky sexual behaviors such as unprotected sex, multiple sexual partners, prolonged and traumatic sex, and risky injections [17-19]. In this study alcohol users were found to be almost six times more likely to have HIV sero-positive result than non-users of alcohol and this might be due to the fact that alcohol drinking increases sexual desire which might be responsible for their exposure to HIV infection by having unprotected sex and multiple sexual partners. This finding is supported by similar study in Addis Ababa [16].

Regarding the association of education level with HIV infection in this study, those who do not have education and those who have primary education level were three times more likely to be infected with HIV those whose education level is secondary and above. This is in line with the fact that people that are more knowledgeable could take care of HIV infection, as they easily understood both the transmission and prevention methods. This finding is similar with the result obtained from another study where people with primary education level or bellow were found to be 2.69 times more likely to be infected with HIV compared with those who have education level of secondary and above [3].

With regard to gender in this study, females were found to be two times more likely to be infected with HIV than males and this finding is supported by a previous study where females were almost three times more likely to be infected with HIV than males [3]. The possible explanation could be that girls are at a much greater risk at early ages because of both biological and cultural factors such as early age at sexual debut, early marriage, sexual abuse and violence.

### Limitation

Using face-to-face interviews for sensitive issues can invite social desirability bias and therefore underestimate sexual activity. Being a case control study recall bias might have been introduced but we have attempted to reduce it by reducing the age interval of the study participants (youths of age between 15–24 years). The study is health facility based and therefore precludes generalization to all youths in Ethiopia indicating a need for further study using a more representative sample of youths in the country. Despite this limitation, the study provides useful information that will inform health service planners to design a strategy for delaying age at first sex and for the prevention of HIV in Ethiopia.

## Conclusion

In conclusion, the prevalence of early sexual initiation in this study was high and the first sexual intercourse was unplanned. Being unemployed, alcohol and khat user and not having comprehensive knowledge on HIV/AIDS were independent predictors of early sexual initiation. In addition, the prevalence of alcohol and khat use was higher among HIV sero-positive youths. Age, sex, education level, marital status and alcohol use were found to be independent predictors of HIV infection. Strategies should be designed to control the use of substances like alcohol and khat, which were found to be responsible for the spread of HIV infection and early sexual initiation in this study. There should be law enforcement from the government of Ethiopia to discourage the flourishing advertisement of alcoholic drinks in the mass media since alcohol consumption is not legally prohibited in Ethiopia and there are no age limits on alcohol drinking.

## Competing interests

The authors declare that they have no competing interests.

## Authors’ contributions

MT was investigator, involved in proposal writing, designing, and recruitment and training of supervisors and data collectors, analysis and write-up and in all stages of the project implementation. He did most of the analysis and write up of the paper. GA contributed in the designing of the methodology, recruitment and training of supervisors and data collectors and involved in designing of project proposal, design of questionnaires, supervision and involved in the final approval of the paper. Both authors read and approved the final manuscript.

## Pre-publication history

The pre-publication history for this paper can be accessed here:

http://www.biomedcentral.com/1472-698X/13/10/prepub
